# The Immobilization of Soil Cadmium by the Combined Amendment of Bacteria and Hydroxyapatite

**DOI:** 10.1038/s41598-020-58259-1

**Published:** 2020-02-10

**Authors:** Xiaoxi Zeng, Hong Xu, Jijie Lu, Qimin Chen, Wen Li, Ling Wu, Jianxin Tang, Liang Ma

**Affiliations:** College of Life Sciences and Chemistry, Hunan University of Technology, Key Laboratory of Biological, Nanomaterials and Devices, Hunan, 412007 P.R. China

**Keywords:** Non-model organisms, Environmental impact

## Abstract

The remediation of heavy metal-contaminated soils has attracted increased attention worldwide. The immobilization of metals to prevent their uptake by plants is an efficient way to remediate contaminated soils. This work aimed to seek the immobilization of cadmium in contaminated soils via a combination method. Flask experiments were performed to investigate the effects of hydroxyapatite (HAP) and the *Cupriavidus* sp. strain ZSK on soil pH and DTPA-extractable cadmium. Pot experiments were carried out to study the effects of the combined amendment on three plant species. The results showed that HAP has no obvious influence on the growth of the strain. With increasing concentrations of HAP, the soil pH increased, and the DTPA-extractable Cd decreased. Via the combined amendment of the strain and HAP (SH), the DTPA-extractable Cd in the soil decreased by 58.2%. With the combined amendment of the SH, the cadmium accumulation in ramie, dandelion, and daisy decreased by 44.9%, 51.0%, and 38.7%, respectively. Moreover, the combined amendment somewhat benefitted the growth of the three plant species and significantly decreased the biosorption of cadmium. These results suggest that the immobilization by the SH combination is a potential method to decrease the available cadmium in the soil and the cadmium accumulation in plants.

## Introduction

Soil is the main sink for metals and acts as a barrier to prevent their entry into the food chain^1^. However, human activities such as mining and metal smelting have gradually transferred many toxic metals from the earth’s crust to the environment, resulting in the spread and contamination of heavy metals^2–4^. In recent decades, heavy metal pollution has become a serious problem worldwide. In China, it was reported that 13.33% of all soil samples collected from 6.3 million square kilometres of land were polluted with high levels of metals^5^. Cadmium pollution has been detected in all agricultural areas. Cadmium is chemically stable and does not undergo chemical or microbial degradation. Moreover, cadmium is not an essential element for living organisms. Generally, it is regarded as the most toxic among heavy metals^6^. Cadmium in the soil harms plant cells by altering metabolic pathways, damaging chloroplasts and mitochondria, and causing oxidative damage to lipids and proteins^7,8^, and cadmium also interferes with the uptake and transport of essential elements, e.g., P and K^9^. Excessive Cd can reduce iron uptake by plants, affecting their photosynthesis^10^. Cadmium can also accumulate in plants and animals and thus enters the human body through the food chain, threatening health^11,12^. Thus, it is an enormous challenge to remediate heavy metal-contaminated soils worldwide.

Various physical, chemical and biological techniques have been used to remediate heavy metal-contaminated soils^1^. In situ immobilization is generally considered a feasible technique to remediate metal-contaminated soils due to its cost effectiveness and ease of operation^13^. Many researchers have reported on the use of various amendments to immobilize Cd in polluted soils, including phosphates, clay minerals, calcareous materials, and so on^14^. However, the long-term use of a single chemical amendment can alter soil properties, resulting in soil alkalization, soil hardening, microbiologic ecological disturbances, and so on^15^. Thus, identifying combinations of chemical and biological materials to compensate for these shortcomings has been a popular approach with respect to the immobilization of soil metals.

Microorganisms are living materials with various properties. Some bacteria and fungi have been investigated to remediate contaminated soils and water^13,16^. The hybrid bio-nanocomposite (ANHP) can be recognized as a promising soil amendment candidate for effective remediation on the soils^17^. In our previous work, the bacterium *Cupriavidus* sp. ZSK was isolated from heavy metal-contaminated soils^16^. It was indicated that this strain is highly tolerant to five heavy metals (Cd, Cu, Zn, Cr, and Pb) and is a potential microorganism to adsorb cadmium ions^16^. In this work, the effects of strain *Cupriavidus* sp. ZSK and HAP on DTPA-extractable cadmium were studied. The potential application of the combined amendment of this strain and HAP was investigated for the immobilization of soil cadmium.

## Materials and Methods

### Flask experiments

The flask experiments consisted of three parts. The first part involved strain growth in the presence of HAP. HAP was added to LB media in flasks at five different ratios (0, 1%, 2%, 3%, and 4%). The flasks were sterilized and inoculated with *Cupriavidus* sp. ZSK in accordance with normal procedures, after which the bacteria were cultured in a shaker at 180 rpm at 30 °C. According to the growth curve of the strain in previous work, the cells were collected at an interval of 3 h during the exponential and stationary phases, after which plate colony counting was implemented. Each treatment involved three replications, for a total of 15 flasks.

Diethylene-triamine-pentaacetic acid (DTPA)-extractable metal is well known as an index of available metals in soils^17^. To investigate the effect of hydroxyapatite on the pH and the DTPA-extractable Cd in the soil, a second experiment was carried out. HAP was added to soils at five different ratios (0, 1%, 2%, 3%, and 4%) and mixed well. The mixtures were then loaded in flasks, and deionized water was added at a soil: water ratio of 2:1 (w/v). Each treatment was conducted in triplicate, for a total of 15 flasks. The flasks were shaken for 2 h to fully soak the soil and then were incubated at room temperature. The soils were sampled at an interval of 2 d and were divided into three portions to analyse the pH and cadmium concentration. The pH was tested by a pH meter PHSJ-4F (Shanghai Thunder magnetic equipment factory), and the cadmium was determined by ICP-MS.

In the third part, the combination of the strain and HAP was evaluated, which was denoted as SH. Specifically, the effects of SH on the pH and DTPA-extractable Cd of soil were studied. There were flasks with soil as described above, and they were established into four groups: Group A was the control flask experiment without HAP and the strain; group B was the flask experiment with 3% HAP; group C was the flask experiment with the strain (10^8^ cells/mL); and group D was the flask experiment with the SH. Each group was replicated three times, for a total of 12 flasks.

The immobilization efficiency was calculated by the following formula:

1 $${\rm{Immobilization}}\,{\rm{efficiency}}\,( \% )=({\rm{a}}-{\rm{b}})/{\rm{a}}\,\ast \,100 \% $$

where a (mg/kg) is the DTPA-extractable Cd of the control group (A) and b (mg/kg) is the DTPA-extractable Cd of the groups with SH (B/C/D).

### Pot experiments

**Figure 1:**
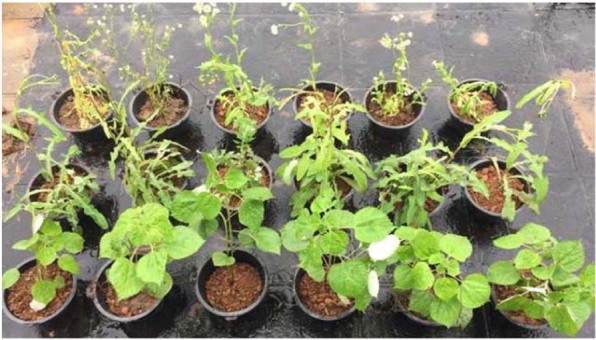
The pot experiment. Each species included two groups: one was the control group without SH, and the other was the treatment group with SH, including 3% HAP (w/w) and 10^8^ cells/g of the *Cupriavidus* sp. ZSK strain. The pots were arranged randomly under natural light. The plants were watered when needed and harvested after 45 d. Each group was replicated three times.

Ramie, dandelion, and daisy are common plant species in polluted areas near the Zhuzhou smelter. Pots 15 cm in height with a top diameter of 23.5 cm and a bottom diameter of 16 cm were used in the experiments. A total of 1.5 kg of cadmium-polluted soil was added to each pot. The seedlings of the plants from unpolluted soils were transplanted into pots. Each species included two groups: one was the control group without SH, and the other was the treatment group with SH, including 3% HAP (w/w) and 10^8^ cells/g of the *Cupriavidus* sp. ZSK strain. The pots were arranged randomly under natural light (Fig. 1). The plants were watered when needed and harvested after 45 d. Each group was replicated three times.

### Cadmium determinations

Cadmium was extracted by DTPA solution at a soil: water ratio of 1:5 (w/v). The samples were then shaken at 180 rpm for 2 h on a thermostatic oscillator at 25 °C. The suspensions were centrifuged at 8000 rpm for 30 min and then filtered with a 0.45-μm membrane filter. The soil pH was determined in 1:2.5 soil: water suspensions after sharking over 0.5 h with a combination pH electrode. The soil samples should be digested in a solution consisting of HF: HClO_4_: HCl: HNO_3_ at a ratio of 1:1.6:2:4. The concentration of Cd in the extracts was tested by ICP-MS.

The potted plants were harvested after 45 d. First, the plants were collected, shaken to remove the soil particles and then weighed by an electronic scale. They were then thoroughly washed with tap water followed by demineralized water. The plant samples were ground to powder after naturally air drying to a constant weight. The soil samples were digested in a solution containing HNO_3_:HClO_4_ at a ratio of 6:1. The concentrations of Cd in the extracts were tested by ICP-MS

### Statistical analysis

The data were subjected to one-way ANOVA using SPSS 17.0 software. At least three independent replicates of each sample and determination were tested, and mean values and respective standard deviations were calculated.

## Results and Discussion

### Soils, strains, and HAP

**Figure 2:**
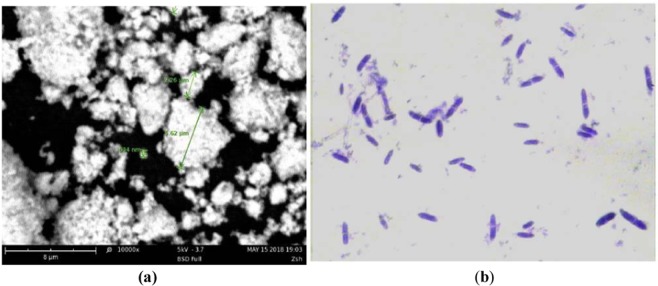
HAP and the bacterial strain: (**a**) SEM image of micro-nanometer-sized HAP particles; (**b**) crystal violet staining of the strain *Cupriavidus* sp. ZSK.

The cadmium-contaminated soil (pH of 6.84, TOC pf 0.952%, total N of 1.73 g/kg, total P of 1.16 g/kg, cation exchange capacity of 0.039 cmol/kg; 37.8% sand, 38.7% silt, 23.4% clay) was collected from gardens near the Zhuzhou Smelter in Hunan Province, China (113°4′23.46″E, 27°51′54.3″N)^18^. It is classified as Ferralsols, according to the World Reference Base for soil resources (WRB)^19^. The soil was cleared of plant debris and stones, air dried at room temperature, and then ground to a particle size 2 mm for the experiments. The total cadmium was 13.82 mg kg^−1^, and the DTPA-extractable Cd was 5.73 mg/kg. *Cupriavidus* sp. ZSK was isolated in our previous work. The bacterium was cultured and observed by optical microscopy to test its activity. HAP was supplied by Shanghai Hualan Chemical Technology Company. HAP is a ultra-fine powder with micron-nanometer dimensions (Fig. 2(a)). The Ca_10_ (PO_4_)_6_(OH) _2_ was ≥99.5%, and the heavy metals were ≤1 ppm; the pH was 8.10.

### The effects of HAP on the bacterial strain

**Figure 3:**
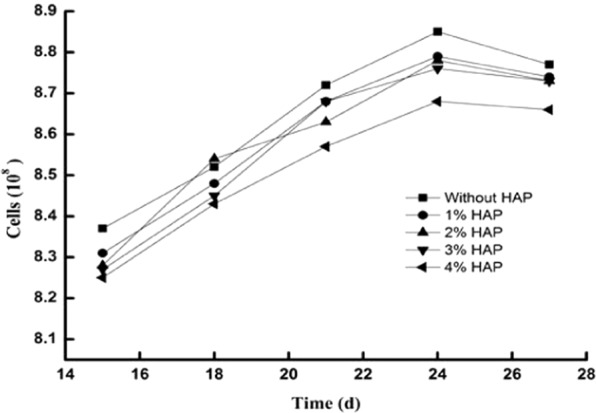
The effects of HAP on the growth of the strain. HAP was added to LB media in flasks at five different ratios (0, 1%, 2%, 3%, and 4%). The flasks inoculated with *Cupriavidus* sp. ZSK were cultured in a shaker at 180 rpm at 30 °C.

The bacterial strain *Cupriavidus sp*. ZSK was cultured in LB media. Crystal violet staining was used to confirm its morphology. Figure 1(b) show that the strain is a rod-shaped bacterium, which means these bacteria were not contaminated with other bacteria. Microorganisms are often affected by the addition of soil amendments; for example, sepiolite and sodium alginate can increase soil microbial biomass and diversity^20,21^, nano-silver is highly cytotoxic and is genetically toxic to nitrifying bacteria^22^, and soil bacterial diversity varies under the addition of HAP^23^. Therefore, according to the growth curve in previous works, the effect of HAP on the strain growth was investigated. Figure 3 shows that without HAP, the peak growth was 8.85 × 10^8^ cells. In flasks with 1%, 2%, and 3% HAP, the peaks were similar to those of the control. With 4% HAP, the growth peak was 8.68 × 10^8^cells, which was slightly lower than that of the control. In all groups, the peaks occurred at 24 h, suggesting that 1–3% HAP has little effect on the growth of the strain.

### The effects of HAP on the pH and DTPA-extracted Cd

**Figure 4:**
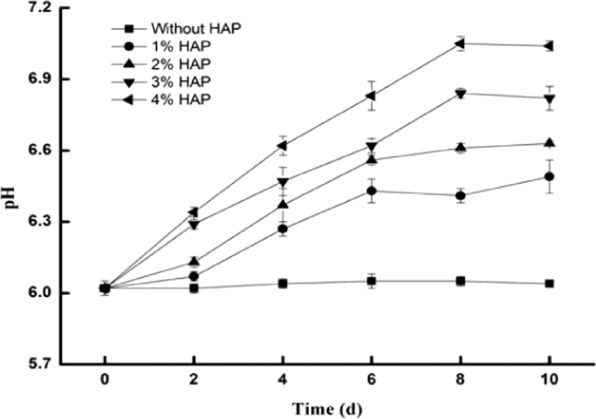
The effects of HAP on the soil pH. HAP was added to LB media in flasks at five different ratios (0, 1%, 2%, 3%, and 4%). The flasks inoculated with *Cupriavidus* sp. ZSK were cultured in a shaker at 180 rpm at 30 °C. Bars represent mean ± SD.

The time course of the soil pH inoculated with HAP is shown in Fig. 4. In the control experiment without HAP, the pH of the soil was relatively stable, ranging from 6.02 to 6.05. In the other groups with HAP, the soil pH increased obviously. With 1, 2, 3, and 4% HAP, the soil pH increased from 6.02 to 6.49, 6.63, 6.82, and 7.04, respectively. In all groups, the pH reached stabilized on the 8th day.

**Figure 5:**
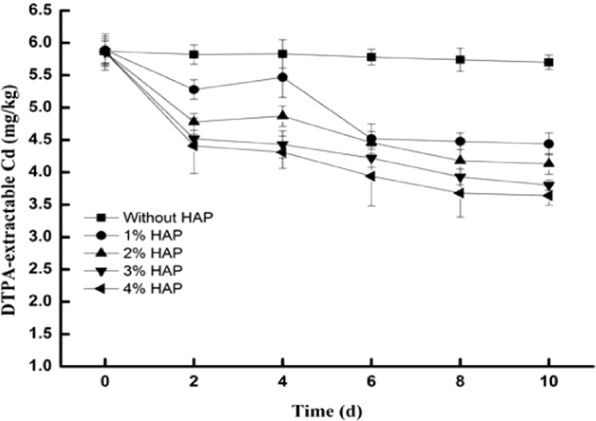
The effects of HAP on the DTPA-extracted Cd. HAP was added to LB media in flasks at five different ratios (0, 1%, 2%, 3%, and 4%). The flasks inoculated with *Cupriavidus* sp. ZSK were cultured in a shaker at 180 rpm at 30 °C. Bars represent mean ± SD.

The DTPA-extracted Cd in the soils is presented in Fig. 5. In the control soils, the cadmium concentration changed slightly, ranging from 5.70 to 5.87 mg/kg. With increasing HAP, the DTPA-extracted Cd decreased gradually. In the flasks with 1, 2, 3, and 4% HAP, the DTPA-extracted Cd decreased by 24.62, 29.53, 35.10, and 37.89%, respectively.

At high pH, mobile cadmium might become immobile. However, an excessively high pH can alter soil properties, which influence soil ecology and plant growth. In general, the increase in pH should be better at values more greater than 1. For the above reason, the HAP concentration of 3% was selected for the following experiments.

### The effects of SH on the pH and DTPA-extractable Cd in flask experiments

As shown in Table 1, the effects of SH on the pH and DTPA-extractable Cd were investigated. The soil pH of group A (without SH) was 6.02. Compared with that of the control group (A), the pH of groups B (with HAP) and D (with SH) obviously increased, reaching 6.75 and 6.77, respectively. In group C (with the strain), the pH was 6.05, which had slightly increased. These results suggested that the strain has no notable effect on the pH and that the effect of the SH on soil pH is dependent on HAP. The statistical analysis showed that the pH of groups B and D were significantly different from the pH of the control group (A) (*p* < 0.05), while that of group C was not (*p* = 0.91).

**Table 1:** The combined effects of the bacterial strain and HAP on soil and cadmium.

Groups	pH (F = 168.14; df = 3)	DTPA-extractable Cd (mg/kg; F = 55.53; df = 3)	Immobilization efficiency (%)
A	6.02 ± 0.04b	5.85 ± 0.43a	—
B	6.75 ± 0.05a	3.92 ± 0.25b	33.0%
C	6.05 ± 0.08b	4.76 ± 0.32b	18.6%
D	6.77 ± 0.03a	2.45 ± 0.31c	58.2%

Group A represents the control flask experiment without HAP and the strain; group B represents the flask experiment with 3% HAP; group C represents the flask experiment with the strain (10^8^ cells/mL); and group D represents the flask experiment with the SH. Each group was replicated three times. According to ANOVA (Tukey’s test), there is no significant difference in the value of the same letter within a single column at *p* < 0.05 (Significant at 95% confidence interval, F: statistics, df: degrees of freedom).

Table 1 show that the DTPA-extractable Cd of groups B, C, and D were lower than that of the control group (A). The DTPA-extractable Cd of group A was 5.85 mg/kg, and that of groups B, C, and D was 3.92 mg/kg, 4.76 mg/kg, and 2.45 mg/kg, respectively. The statistical analysis showed that the DTPA-extractable Cd of groups B, C, and D was significantly different that of the control group (A) (*p* < 0.05). This means that HAP, the bacterial strain, and SH reduced the DTPA-extractable Cd by 33.0%, 18.6%, and 58.2%, respectively. The SH presented the best capability of decreasing the DTPA-extractable Cd in the soil.

### The effects of SH on the biomass and cadmium accumulation of the plants

**Figure 6:**
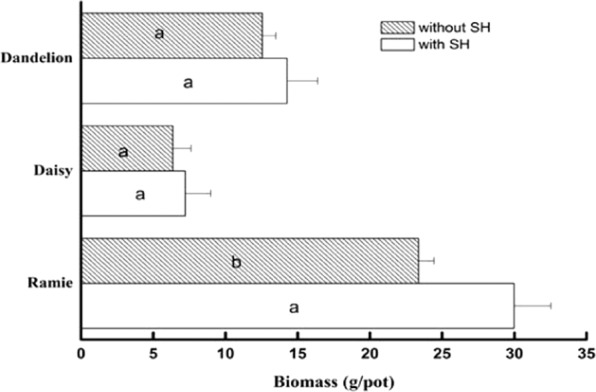
The effects of SH on the biomass of plants. Bars represent mean ± SD. The different letters within the columns of three plants indicate significant differences, while the same letters indicate no significant differences (*p* < 0.05).

**Figure 7:**
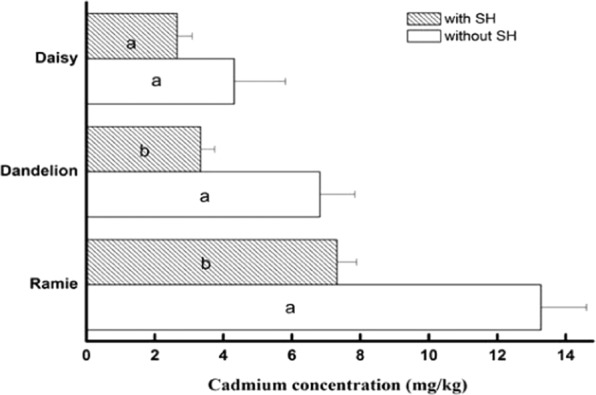
The effects of SH on cadmium accumulation in plants. Bars represent mean ± SD. The different letters within the columns of three plants indicate significant differences, while the same letters indicate no significant differences (*p* < 0.05).

The potted plants were harvested. The biomass values are shown in Fig. 6. All of the biomass values of the plants treated with SH were much greater than those of the control plants. The biomass of dandelion and daisy increased slightly in the treatments with SH addition compared to the control treatment (*p* > 0.05). In ramie, the biomass significantly increased in the treatments with SH addition compared to the control treatment (*p* = 0.01), increasing by 28.3%. This suggests that HAP not only is harmless but also somewhat benefits the growth of the plants. However, compared with that in the non-SH groups, the cadmium accumulation in all plants in the other groups decreased (Fig. 7). In the dandelion group, cadmium accumulation decreased from 6.82 mg/kg to 3.34 mg/kg. This decrease is the maximum among the three plant species, which reached 51.0%. The cadmium accumulation decreased by 44.9% and 38.7% in the ramie and daisy groups, respectively. The statistical analysis showed that the cadmium accumulation in dandelion and ramie significantly decreased (*p* < 0.05) but that the accumulation in the daisy did not (*p* = 0.14). This means that SH can significantly reduce the bioavailable cadmium and induce plants to absorb cadmium from the soil.

Immobilization is considered a potentially reliable, cost-effective technique for the reclamation of metal-contaminated soils^5^. At present, some chemical amendments have been investigated and applied for the immobilization of heavy metals. Phosphates and their derived materials are considered effective amendments for contaminated soils^24^. HAP is a typical inorganic chemical amendment that immobilized heavy metals^25^. Generally, the primary mechanisms of HAP for metal immobilization are adsorption, precipitation, cation exchange, and surface complexation, and involved processes as the follows^26–28^ (Eqs. 2–4): HAP is dissolved in acidic soil, then Cd ions are adsorbed on the surface of HAP to form phosphate precipitates. Cd^2+^ with Ca^2+^ ion exchange resulting in the formation of a Cd-containing HAP^17,29–31^^.^

2 $${{\rm{Ca}}}_{10}{({{\rm{PO}}}_{4})}_{6}{({\rm{OH}})}_{2}+14{{\rm{H}}}^{+}\to 10{{\rm{Ca}}}^{2+}+6{{\rm{H}}}_{2}{{\rm{PO}}}_{4}^{-}+2{{\rm{OH}}}^{-}$$

3 $$10{{\rm{Cd}}}^{2+}+6{{\rm{H}}}_{2}{{\rm{PO}}}_{4}^{-}+2{{\rm{OH}}}^{-}\to {{\rm{Cd}}}_{10}{({{\rm{PO}}}_{4})}_{6}{({\rm{OH}})}_{2}+12{{\rm{H}}}^{+}$$

4 $${{\rm{Cd}}}_{10}{({{\rm{PO}}}_{4})}_{6}{({\rm{OH}})}_{2}+{{\rm{\lambda }}\text{Cd}}^{2+}\to ({{\rm{Cd}}}_{{\rm{\lambda }}}{{\rm{Cd}}}_{10+{\rm{\lambda }}}^{-}){({{\rm{PO}}}_{4})}_{6}{({\rm{OH}})}_{2}+{{\rm{\lambda }}\text{Ca}}^{2+}$$

Moreover, in this work, the cadmium contaminated soil is from Zhuzhou city nearby the Xiangjiang River, in southern China. The soil is slight acid, marked as ferralsol. In the area, weaker atmospheric dispersion, higher humidity and more sunlight favor the formation of acid rain^32^. Acid rain could cause soil acidifying and damages the soil construction. Introduction of the alkaline matters in soils can weaken soils acidity^1^. Thus, HAP can not only immobilize metals but also benefiting the soils stabilizations.

It is well known that HAP can increase the pH to form stable metal-phosphate precipitates in heavy metal-contaminated soils^5,33^. With a high specific surface area and reactivity, nano-hydroxyapatites (n-HAPs) are more effective than bulk HAP particles for immobilizing metals^13^. Considering that n-HAP particles tend to self-agglomerate during the process of application, the micro-nanometer HAP was used in this work. In this study, Figs. 4 and 5 show that the pH obviously increased with increasing HAP in the soils, and the DTPA-extracted Cd decreased. The micro-nanometer HAP can increase the soil pH to reduce the availability of heavy metals^34^. This result is consistent with some reports^27,35,36^.

Some microorganisms also can immobilize heavy metal ions. Li *et al*.^37^ reported that the *Rhizobium pusense* KG 2 can immobilize Cd^2+^ from soil. Yang *et al*.^17^ studied that hybrid bio-nanocomposites of fungal hyphae and nano-hydroxyapatites immobilize Cd and Pb in contaminated soils suggested it can be recognized as a promising soil amendment candidate. In our previous study, the bacteria strain *Cupriavidus* sp. ZSK can tolerant to and adsorb heavy metals^16^. When the bacterial strain *Cupriavidus* sp. ZSK was introduced with HAP in the flask experiments, the soil pH did not change obviously, but the DTPA-extractable Cd decreased from 3.35 mg/kg to 2.45 mg/kg. This suggests that the efficiency of immobilizing cadmium by the combined amendment is obviously better than that by the strain or HAP. The adsorption ability of the *Cupriavidus* sp. strain contributes to the stabilization of cadmium ions in the soil. Compared with that in other similar studies, the efficiency of immobilization by SH is moderate to high^38–41^. The pot experiments suggest that SH obviously decreases the cadmium accumulation in the three plant species, which means that the bioavailable cadmium decreased in the soil. Moreover, SH is benefit to plant growth. Thus, the combination of the *Cupriavidus* sp. ZSK strain and HAP is considered a potential amendment for the immobilization of soil Cd. The detail relationship and interactive mechanism of the strain and HAP need further investigate.

## Conclusions

Micro-nanometer HAP caused the soil pH to increase, and DTPA-extractable cadmium decreased significantly. The efficiency of immobilizing cadmium by the combined amendment of the *Cupriavidus* sp. ZSK strain and HAP was obviously higher than that by the strain or HAP alone. The combined amendment somewhat benefits plant growth and can significantly decrease cadmium accumulation in plants. Thus, the combination of the bacterial strain *Cupriavidus* sp. ZSK and HAP represents a potential method to decrease available cadmium in the soil and cadmium accumulation in plants.
